# Abnormal static and dynamic functional network connectivity of the whole-brain in children with generalized tonic-clonic seizures

**DOI:** 10.3389/fnins.2023.1236696

**Published:** 2023-08-21

**Authors:** Yongxin Li, Yun Ran, Qian Chen

**Affiliations:** ^1^Formula-Pattern Research Center, School of Traditional Chinese Medicine, Jinan University, Guangzhou, China; ^2^Department of Pediatric Neurosurgery, Shenzhen Children’s Hospital, Shenzhen, China

**Keywords:** generalized tonic-clonic seizure children, resting-state fMRI, independent component analysis, static functional connectivity, dynamic functional connectivity

## Abstract

**Introduction:**

Generalized tonic-clonic seizures (GTCS) are a subtype of generalized seizures exhibiting bursts of bilaterally synchronous generalized spike-wave discharges. Numerous neuroimaging studies have reported aberrant functional activity and topological organization of brain network in epilepsy patients with GTCS, but most studies have focused on adults. However, the effect of GTCS on the spatial and temporal properties of brain function in children remains unclear. The present study aimed to explore whole-brain static (sFC) and dynamic functional connectivity (dFC) in children with GTCS.

**Methods:**

Twenty-three children with GTCS and 32 matched healthy controls (HCs) were recruited for the present study. Resting-state functional magnetic resonance imaging (MRI) data were collected for each subject. The group independent component analysis method was used to obtain independent components (ICs). Then, sFC and dFC methods were applied and the differences in functional connectivity (FC) were compared between the children with GTCS and the HCs. Additionally, we investigated the correlations between the dFC indicators and epilepsy duration.

**Results:**

Compared to HCs, GTCS patients exhibited a significant decrease in sFC strengths among most networks. The K-means clustering method was implemented for dFC analysis, and the optimal number of clusters was estimated: two discrete connectivity configurations, State 1 (strong connection) and State 2 (weak connection). The decreased dFC mainly occurred in State 1, especially the dFC between the visual network (VIS) and somatomotor network (SMN); but the increased dFC mainly occurred in State 2 among most networks in GTCS children. In addition, GTCS children showed significantly shorter mean dwell time and lower fractional windows in stronger connected State 1, while GTCS children showed significantly longer mean dwell time in weaker connected State 2. In addition, the dFC properties, including mean dwell time and fractional windows, were significantly correlated with epilepsy duration.

**Conclusion:**

Our results indicated that GTCS epilepsy not only alters the connectivity strength but also changes the temporal properties of connectivity in networks in the whole brain. These findings also emphasized the differences in sFC and dFC in children with GTCS. Combining sFC and dFC methods may provide more comprehensive understanding of the abnormal changes in brain architecture in children with GTCS.

## 1. Introduction

Generalized tonic-clonic seizures (GTCS) are a subtype of generalized seizures exhibiting bursts of bilaterally synchronous generalized spike-wave discharges (2.5–5 Hz) (Ji et al., 2014). During such seizures, patients usually exhibit symptoms of rigid stiffening of the limbs, violent muscle contractions of the body, and loss of consciousness (Zhang et al., 2011). People with GTCS also show various cognitive impairments between seizures, such as deficits in attention, memory, and executive function (Paige and Cavanna, 2013). Additionally, patients with GTCS do not exhibit focal anatomical brain lesions on routine MRI scans. Because of these cognitive impairments and anatomical characteristics, substantial effect has been made to understand the neural mechanisms underlying GTCS (Zhang et al., 2011; Kim et al., 2014; Wang et al., 2018; Allen et al., 2020; Li et al., 2020a).

Neuroscience research has indicated that epilepsy can be regarded as a functional brain network disorder associated with excessive synchronization of large neuronal populations (Engel et al., 2013; Royer et al., 2022). It disrupts the balance between excitatory and inhibitory activities (Fisher et al., 2017a). With the development of non-invasive functional magnetic resonance imaging (fMRI) techniques, considerable progress has been made in understanding the neurological pathogenesis of GTCS. Previous high-resolution structural image analysis of GTCS patients have revealed widespread and subtle anatomical abnormalities in cortical and subcortical regions (Huang et al., 2011; Kim et al., 2013; Zhou et al., 2015; Liao et al., 2016; Ke et al., 2017). These abnormalities were mainly located in the thalamus, hippocampus, cingulate cortex, cerebellum, and frontal lobe (Ciumas and Savic, 2006; Huang et al., 2011; Zhou et al., 2015; Allen et al., 2020). The abnormal discharges of GTCS not only alter brain morphometry but also affect intrinsic brain activity and large-scale brain network properties in the interictal period. Resting-state fMRI studies of GTCS patients have revealed abnormal intrinsic brain activity and interhemispheric communication in the thalamus, temporal pole, angular gyrus and prefrontal cortex (Zhong et al., 2011; Ji et al., 2014; Wang et al., 2014; Yang et al., 2014; Wang et al., 2018). From the perspective that epilepsy represents a brain network disorder, the functional and structural brain has alterations have also been studied with the graph theory approach in both adults (Zhang et al., 2011; Liao et al., 2013; Li et al., 2016) and children (Li et al., 2020a,b, 2022b) with GTCS.

Moreover, resting-state fMRI has become a popular technique for studying epilepsy. This technique provides an effective way to quantify the intrinsic functional organization of the brain. To assess functional connectivity (FC), interregional synchronization is examined by measuring the correlation of brain signals in resting-state fMRI data. Given that the association of epilepsy with abnormal FC in several resting-state networks has gained widespread acceptance (Tracy and Doucet, 2015; Royer et al., 2022), there is growing research interest in detecting the changes in FC of resting-state fMRI data in GTCS patients (Wang et al., 2011; Kim et al., 2014; Ke et al., 2017; Li et al., 2017; Wang et al., 2019; Hsieh et al., 2022). For example, one recent study using the seed-based FC method revealed decreased FC of the cingulated cortex in GTCS patients (Ke et al., 2017). The abnormalities in FC might be related to the decreased volume of the cingulate cortex. Recent resting-state fMRI studies have reported altered FC in the default mode network (DMN), thalamocortical network, sensorimotor network (SMN), and ventral attention network (VAN) (Wang et al., 2011; Wang et al., 2019; Li et al., 2020a). In particular, the DMN showed more significantly and frequently altered FC than other resting state networks in GTCS epilepsy patients (Song et al., 2011; Parsons et al., 2020). Overall, these previous reports examined the changes in network FC of patients with GTCS, which may be related to clinically relevant phenomena.

Notably, the aforementioned functional imaging studies have employed static FC (sFC) to investigate the intrinsic brain architecture, which assumes that there is no fluctuation of brain signals throughout the entire scan. Even though sFC has been used successfully to determine brain abnormalities in GTCS, this method overlooks the temporal properties of FC. Recent studies have proven that brain FC in the resting state is indeed dynamic (Fox et al., 2005; Rolls et al., 2021). Since 2010, dynamic FC (dFC) methods have been developed to examine time-varying network connectivity in resting-state fMRI data (Chang and Glover, 2010; Allen et al., 2014). The advantages of this methodology have been demonstrated in recent studies, and this method has been applied in epilepsy research (Liu et al., 2017; Jia et al., 2020; Kowalczyk et al., 2020; Yang et al., 2021). For instance, a dFC study in GTCS patients demonstrated that adult patients had state-specific dFC disruptions and significant changes in temporal metrics (Liu et al., 2017). The majority of aberrant dFC was in the DMN, as confirmed by a recent study that reported that general DMN abnormalities were found across epilepsy types (Yang et al., 2021). Another dFC study on GTCS patients also found that adult patients showed increased variability of FC in regions of the DMN, VAN, and motor-related areas (Jia et al., 2020). FC variability between the DMN and cognition-related networks showed a significant increase. In our previous studies, we applied the dFC method in children with GTCS to explore FC characteristics of specific networks (Li et al., 2022a,c). Children with GTCS showed both an increase and decrease in dFC within the DMN and the thalamocortical network. Although these previous studies demonstrated significant brain abnormalities in terms of activity and connectivity patterns in GTCS patients, most of these previous studies were conducted in adults. Only a few studies have focused on neuroimaging changes in children with GTCS. Comparing the neuroimaging results among these previous studies, we observed that the main findings are not fully consistent between children and adults with GTCS (Song et al., 2011; Li et al., 2016, 2020a,2022a; Liu et al., 2017). Children with GTCS have shown some specific changes in their brain activity and topological properties of their brain network compared with adult patients. In addition, the brain’s dynamic activity changes during the resting state in children with GTCS mainly occurred in the DMN or thalamocortical network (Li et al., 2022a,c). The dynamic interaction among whole-brain functional networks in children with GTCS is still unclear. Investigation of whole-brain dFC would help to fully capture the functional abnormalities and to provide more important information about the neural mechanisms underlying GTCS in children.

To fully explore abnormalities in brain functional network connectivity in children with GTCS, resting-state fMRI was used in the present study to evaluate the sFC and dFC of whole-brain networks. Our hypotheses were as follows: (1) children with GTCS would show abnormal sFC and dFC of the whole brain network and (2) altered dFC would provide more information than sFC, and the temporal properties of dFC would correlate with epilepsy duration.

## 2. Materials and methods

### 2.1. Subjects

Twenty-three children with GTCS (10 females and 13 males, mean age: 68.20 ± 45.22 months) were recruited from Shenzhen Children’s Hospital. All patients with epilepsy were diagnosed with GTCS based on seizure symptoms and scalp-EEG information, consistent with the current International League Against Epilepsy seizure type classifications (Fisher et al., 2017b). Patients were included in the study based on the following criteria: (1) exhibiting typical clinical symptoms of GTCS, such as limb movement, loss of consciousness during seizures, and no partial seizures; (2) exhibiting specific patterns of electrophysiological activity recorded by electroencephalography (generalized spike-and-wave or poly-spike-wave discharges); and (3) not exhibiting abnormalities on a diagnostic MRI. Each patient was treated with at least one antiepileptic drug, including topiramate, valproic acid, oxcarbazepine, and/or levetiracetam. All patients were seizure-free for at least 2 days before the MRI examination. In addition, thirty-two healthy controls (HCs, 10 females, and 22 males, mean age: 75.30 ± 29.77 months) were recruited. All HCs were interviewed to confirm that they did not have a history of neurological disorders or psychiatric illnesses. During the MRI scan, participants under the age of 4 years were sedated with 10% chloral hydrate to reduce their head movement and enhance the success rate of imaging acquisition (dosage: 50 mg/kg, maximum dose of 1 g). Fifteen participants (9 patients and 6 HCs) in this study were under 4 years of age. The demographic and clinical characteristics of patients and HCs are presented in Table 1.

**TABLE 1:** Demographic and clinical information data of the subjects.

Characteristics	Patient group (Mean ± SD)	Control group (Mean ± SD)	Comparisons
Gender (female/male)	10/13	10/22	*X*^2^ = 0.86 (*p* = 0.35)
Age (month)	68.20 ± 45.22	75.30 ± 29.77	*t* = 0.60 (*p* = 0.55)
Epilepsy duration (month)	35.65 ± 36.21	\	

This study was approved by the Ethical Committee of Shenzhen Children’s Hospital. Written informed consent was obtained from each participant’s parents or legal guardians after the explanation of the study purpose, procedures, possible risks, and discomforts and before imaging.

### 2.2. Data acquisition

MRI data were acquired on a German Siemens Trio Tim 3.0T scanner (MAGNETOM, Germany) with an 8-channel head coil at Shenzhen Children’s Hospital, Shenzhen, China. Foam padding and earplugs were used to minimize head movements and machine noise for all subjects. High-resolution structural three-dimensional (3D) T1-weighted images were obtained using the MPRAGE sequence to cover the entire brain for all subjects: repetition time (TR) = 2,300 ms, echo time (TE) = 2.26 ms, field of view (FOV) = 200 × 256 mm^2^, acquisition matrix = 200 × 256, 160 sagittal slices, slice thickness = 1 mm, and flip angle = 8°. Resting-state functional MRI were acquired for each subject using an echo-planar imaging sequence with the following parameters: TR = 2,000 ms, TE = 30 ms, FOV = 220 × 220 mm^2^, matrix size = 94 × 94, slice thickness = 3 mm, flip angle = 90°, number of total volumes = 130, and 36 interleaved axial slices covering the entire brain. During data acquisition, the participants over the age of four were instructed to hold still, close their eyes, avoid thinking about anything in particular, and not fall asleep. To prevent these participants from falling asleep, we observed them throughout the whole scanning process and confirmed that they were awake immediately after image scanning. During the scanning process, T1 images were collected first, and then the resting-state image were collected. During the whole data collection process, no patient had a seizure.

### 2.3. Data preprocessing

The resting-state fMRI data preprocessing was performed using the data assistant software DPABI (Yan et al., 2016). The initial 10 functional images of each subject were discarded to ensure magnetization equilibrium. Subsequently, the remaining volumes were corrected according to the acquisition time delay among different slices and realigned to the first volume to correct for head motion. Excessive motion was defined as more than 3 mm of translation or greater than a 3° rotation in any direction. The mean framewise displacement (FD) was computed by averaging the FD of each participant across the time points. The participants were excluded if the mean FD excluded 0.5 mm or more than 20% of all-time points had FD values exceeding 0.5 mm. No participant was excluded due to excessive motion or excessive FD. Then the mean FD values for all participants were compared between the patients and HCs. No significant differences were found between groups in terms of FD values (GTCS: 0.15 ± 0.09, HC: 0.14 ± 0.12, *T* = 0.35, *p* = 0.73). Subsequently, each subject’s 3D T1-weighted structural images were coregistered to their mean functional images by rigid body transformation. Then, the transformed structural images were segmented by using a unified segmentation algorithm and normalized to Montreal Neurological Institute space by using a 12-parameter non-linear transformation. The transformation parameters were applied to the functional images, and they were resampled to a 3 mm isotropic voxel. The functional images were spatially smoothed with a Gaussian kernel of 6 mm full-width at half maximum.

### 2.4. Group independent component analysis

Group spatial independent component analysis (ICA) was adopted to decompose all preprocessed resting-state data into independent components (ICs) using GIFT software^1^ (Calhoun et al., 2001). Group spatial ICA is a data-driven approach that consists of three stages: dimensionality reduction, IC estimation, and back reconstruction. The preprocessed data from all participants were concatenated into a single dataset. Data reduction involving principal component analysis was conducted to reduce the dimensions of the functional data. ICA was performed to decompose the grouped data into 26 independent components using an Infomax algorithm. This step was repeated 20 times using the ICASSO algorithm to assess the repeatability or stability of ICs. Aggregate spatial maps were estimated as the modes of the component clusters. ICs and time courses for each participant were back-reconstructed. Through back-reconstruction, the individual time courses and spatial maps were obtained.

**FIGURE 1:**
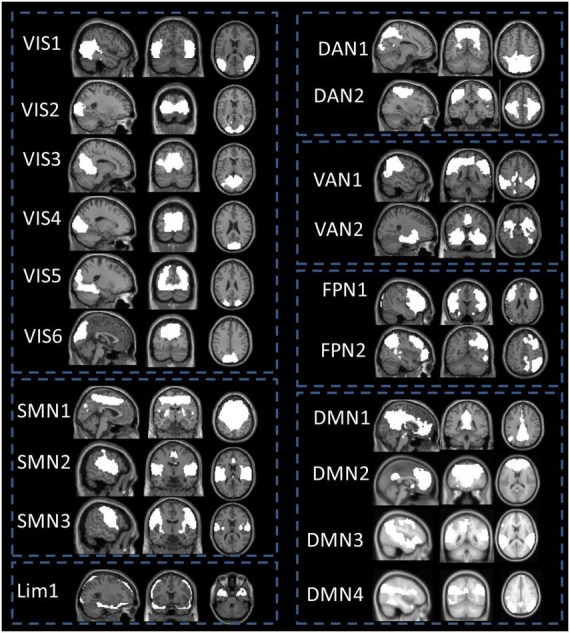
Spatial maps of the 20 independent components grouped into seven different functional domains. Abbreviated labels denote the following networks: Vis, visual network; SMN, somatomotor network; DAN, dorsal attention network; VAN, ventral attention network; Lim, limbic network; FPN, frontoparietal network; DMN, default mode network.

A systematic procedure was used to diagnose the artifacts and identify the functional networks. We used the cortical parcellation maps of the Yeo2011 resting state network^2^ as a template and multiple linear regression as implemented in the spatial sorting function of GIFT, to compare the spatial pattern of each IC with these templates. The functional module of “Component Labeller” in the GIFT toolbox was used to produce a txt file containing a correlation index. Components that had a higher correlation coefficients (larger than 0.2) with these maps of Yeo2011 templates were considered the most related components. The reason for selecting this correlation criterion was based on previous references, in which a reasonable choice of correlation coefficients was studied between ICs from different datasets (Smith et al., 2009; Segall et al., 2012). Previous studies have verified that this threshold can conservatively represent a significance level of *p* < 0.005, corrected (Smith et al., 2009). Some components were excluded from the remainder of the analysis because they were correlated with motion artifacts or spatial maps that included white matter, the ventricular system, or cerebral spinal fluid or because they had irregular time course spectral power. Among the 26 ICs, 20 ICs were acquired by the above process and categorized into seven functional subnetworks based on the Yeo2011 template: the visual network (VIS), SMN, dorsal attention network (DAN), VAN, limbic network (Lim), frontoparietal network (FPN) and DMN (see Figure 1). The subsequent steps were applied to the time courses of these 20 meaningful components to remove physiological and scanner noise: linear detrending, reducing the influence of the Friston 24 realignment parameters using regression and filtering (with a bandpass filter of 0.01–0.1 Hz) (Cordes et al., 2001).

### 2.5. Static functional connectivity analysis

To estimate static FC between the meaningful ICs, the mean time course was first computed by averaging the blood oxygen level-dependent signals over all the voxels within each IC. The correlations of the mean time courses were then computed (Pearson correlation). To normalize the variance in the correlation values, all the resulting correlation coefficients were transformed into a z-score using Fisher’s z-transformation. The normalized correlation values of each pair were regarded as the network edges. A 20 × 20 correlation matrix was generated for each subject.

Then, we used a general linear model with age and sex as nuisance covariates to determine which pair of ICs was significantly different between the groups. The significance threshold was set at *p* < 0.01 to address multiple comparisons in the functional connectivity analysis.

### 2.6. Dynamic functional connectivity analysis

The dFC analysis was performed using a sliding time-window approach in the Dynamic BC toolbox (Liao et al., 2014) (V2.2^3^). The rectangle sliding window size was set to a width of 30 TRs, with a step of 1 TR, resulting in 90 overlapping windows per subject. After that, a k-means clustering algorithm was performed on the windowed correlation matrices from all windows to assess patterns of functional connectivity that reoccurred over time across different participants. We determined the optimal value of k by using the silhouette algorithm. Clustering numbers from 2 to 10 were selected to represent the cluster states. Random initializations were also used in this process to obtain the state cluster centroids. According to the above process, the optimal clustering number was two, namely, State 1 and State 2. The FC matrices of all participants were thus classified into 2 states according to their similarity to the cluster centroids. State transition vectors of all participants across time windows were also derived. For the visualization of the group-specific cluster centroids of the GTCS group and HC group, we also calculated the group-specific cluster centroids by averaging the subject-specific centroids of each group. A two-sample *t*-test was used to compare the connectivity strength of each state (*p* < 0.01) between the HCs and GTCS groups.

**FIGURE 2:**
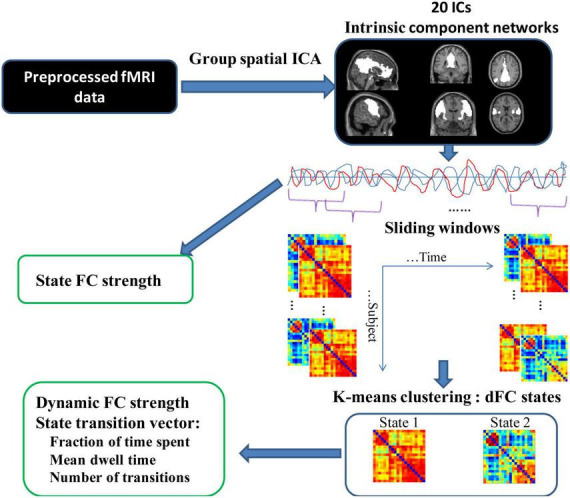
Schematic diagram of the analysis procedure. Resting-state fMRI data were preprocessed and subjected to group spatial ICA resulting in 20 IC networks. Static FC was then estimated. To estimate dFC, a sliding window approach was employed to obtain the subject-specific windowed dFC matrices. K-means clustering was applied to the correlation matrices of all subjects to obtain 2 cluster states. Based on these two states, the state transition vector and dFC strength were obtained for each subject in different states.

Subsequently, the state transition vectors were used to calculate the temporal properties of dFC states: mean dwell time (MDT), fraction of time spent, and number of transitions. The MDT is calculated by averaging the number of consecutive windows belonging to one state, which represents the average duration of time intervals spent in each state. The “fraction of time spent” in each state index is measured as the percentage of time spent in each state out of total time. The “number of transitions” is defined as the number of times subjects switch between different states. Figure 2 shows the schematic diagram of the analysis pipeline. To verify the stability of the dFC results, we also selected a sliding window with a size of 40 TRs and repeated the above analysis. Similar results were obtained by using different window lengths (see Supplementary material). Supplementary Figures 1–3 showed the dFC results of the window length of 40 TRs. Regarding group differences in temporal properties of the dFC state, the significance of group differences in mean dwell time, fraction of time spent, and number of transitions were examined using a two-sample *t*-test (*p* < 0.05) after controlling for age and sex.

### 2.7. Correlation analysis of the relationship between image indices and clinical characteristics

We further calculated the brain-behavior relationships in the patient group. Pearson’s correlation analyses were performed to examine the relationship between the temporal properties of FC states (dwell time, fraction of time spent, and number of transitions) and patient clinical characteristics (epilepsy duration). A threshold of *p* < 0.05 was recognized as statistically significant. During the comparison process, age and sex were controlled.

## 3. Results

### 3.1. Demographic characteristics

The detailed demographic and clinical characteristics of both groups are summarized in Table 1. There were no significant differences between the GTCS patients and HCs in terms of age or sex distribution. Furthermore, information on epilepsy duration was also collected and is listed in Table 1.

### 3.2. Static functional connectivity analysis

**FIGURE 3:**
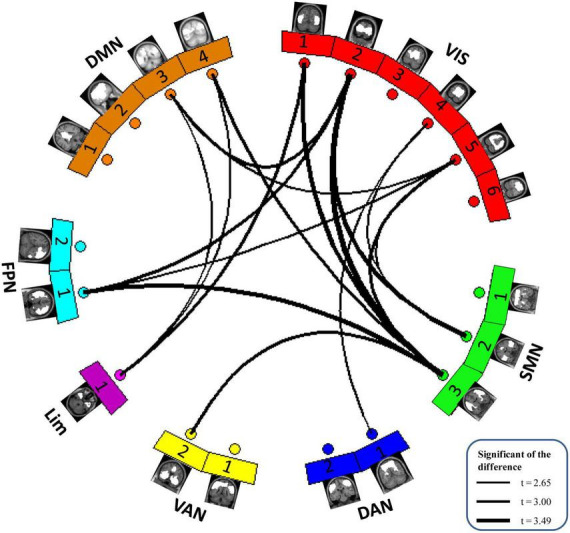
The difference in static functional network connectivity between the two groups in seven functional networks. Compared with the HCs, children with GTCS showed a significant decrease in static FC among the seven functional networks. The thickness of the lines represents the magnitude of FC difference between the two groups.

After group ICA, we grouped these meaningful ICs into seven functional networks based on the Yeo2011 template, namely, the VIS (6 ICs), SMN (3 ICs), DAN (2 ICs), VAN (2 ICs), LIM (1 IC), FPN (2 ICs) and DMN (4 ICs). Detailed spatial maps of ICs identified with group ICA were shown in Figure 1. The results of altered static FC in children with GTCS are shown in Figure 3. Compared with HCs, children with GTCS exhibited significantly decreased static FC between the following networks: VIS-SMN, VIS-DMN, VIS-FPN, VIS-DAN, FPN-SMN, DMN-Lim, and VAN-SMN.

### 3.3. Dynamic functional connectivity state analysis

**FIGURE 4:**
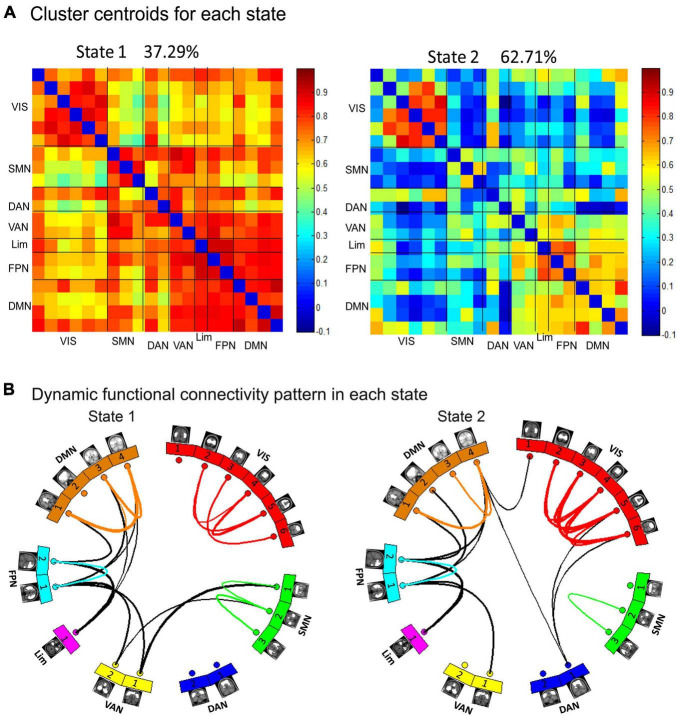
Dynamic functional network connectivity states were identified by clustering analysis. **(A)** The two cluster centroids from all sliding time windows of all subjects are shown along with the percentage of occurrences, which reflects different interregional connectivity patterns in the two states: the State 1 with fewer time windows (37.29%) was mainly characterized by strong interactions and the State 2 with more time windows (62.71%) was mainly characterized by weak interactions. **(B)** In each state, the top 5% of strong connections of each state were retained and are shown in the circular maps.

To examine the recurring patterns of dFC, we identified two patterns of functional connectivity states (State 1 and State 2) that recurred during individual scans and across subjects using a k-means clustering method. These two matrices reflected different interregional connectivity patterns of the two states (Figure 4). State 1 with fewer time windows (37.29%) was mainly characterized by strong positive interregional interactions. State 2 with more frequent time windows (62.71%) was mostly characterized by low dFC.

**FIGURE 5:**
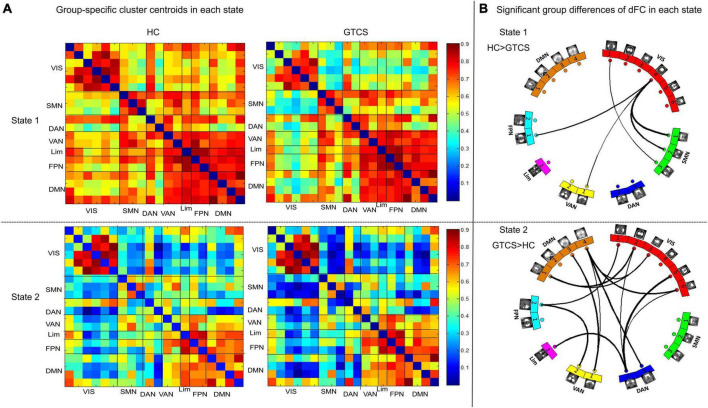
Functional connectivity results in each state. **(A)** Group centroid metrics for each state. **(B)** Children with GTCS had weaker dFC pathways in State 1 and stronger dFC pathways in State 2 than the HC group.

Figures 5A, B demonstrate state- and group-specific cluster centroids obtained by the k-means cluster analysis. In State 1, compared to HCs, GTCS children showed weaker dFC strengths between the following networks: VIS-SMN, VIS-VAN, and VIS-FPN. In State 2, GTCS children exhibited stronger dFC strengths primarily between the following networks: VIS-DMN, VIS-DAN, VIS-FPN, DMN-VAN, DMN-DAN, FPN-VAN, and Lim-DAN.

**FIGURE 6:**
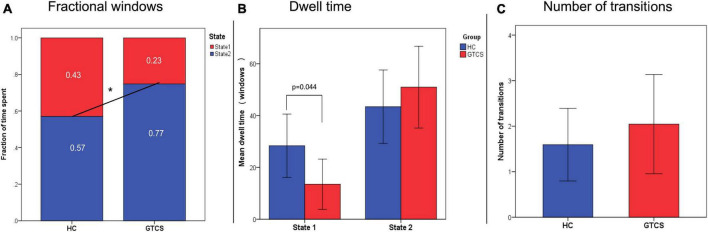
Temporal properties of the dynamic states. **(A)** The mean fractional properties spent in each state differed between the groups. **(B)** The mean dwelling time in State 1 was shorter in GTCS children than in controls. **(C)** The number of transitions between the two groups. *P < 0.05, GTCS, generalized tonic-clonic seizures children; HC, healthy control.

We found a significant group difference in fractional windows (*p* < 0.05), which suggests that children with GTCS exhibited an abnormal proportion of time spent in each state compared to HCs (Figure 6A). In HCs, the total occurrences of State 2 (0.57 ± 0.42) were more frequently observed than State 1 (0.43 ± 0.42). In the patient group, State 1 occurred less frequently (0.23 ± 0.32), and State 2 occurred at a higher rate (0.77 ± 0.32). Thus, in children with GTCS, State 1 occurrence dropped by 0.2, while State 2 presentation increased by 0.2.

As shown in Figure 6B, significant group differences were identified in the MDT of State 1. The MDT in State 1 was significantly shorter in children with GTCS than in healthy controls (HCs: 28.36 ± 33.92, GTCS: 12.55 ± 21.90, *p* = 0.044). This result implied that children with GTCS spent less time in the state with higher connectivity. The MDT in State 2 was higher in children with GTCS but not at significant levels (HCs: 43.42 ± 39.03, GTCS: 52.26 ± 35.93, *p* = 0.403). As shown in Figure 6C, no group differences were found in terms of the number of transitions between states (HCs: 1.59 ± 2.21, GTCS: 2.05 ± 2.52, *p* = 0.492).

### 3.4. Relationship between dFC properties and epilepsy duration

**FIGURE 7:**
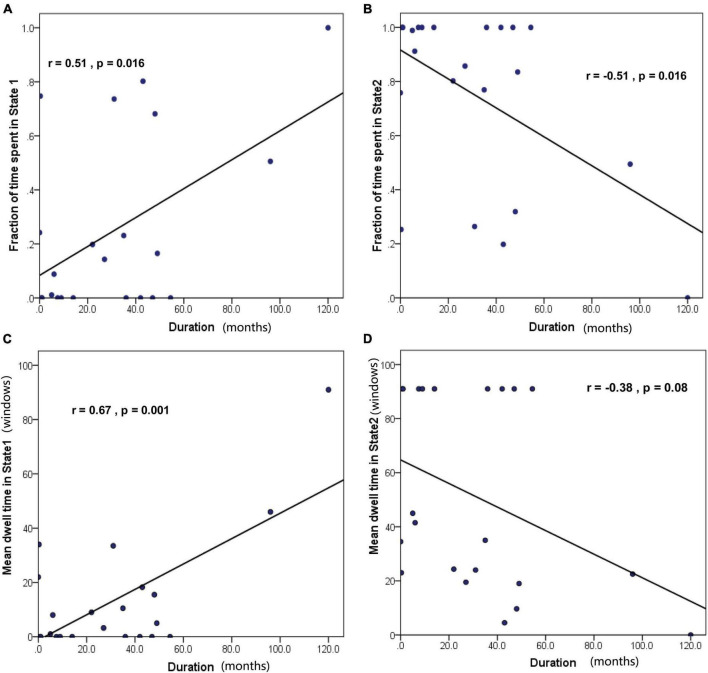
Correlation between the temporal properties of dFC and epilepsy duration in GTCS children. **(A)** The fraction of time spent in State 1 was positively correlated with epilepsy duration. **(B)** The fraction of time spent in State 2 was negatively correlated with epilepsy duration. **(C)** The mean dwell time in State 1 was positively correlated with epilepsy duration. **(D)** The mean dwell time in State 2 was not significantly correlated with epilepsy duration.

Correlation analyses were carried out to test whether dFC properties were associated with epilepsy duration in children with GTCS. Notably, we found that the fraction of time spent in State 1 was positively correlated with epilepsy duration (Figure 7A, *r* = 0.51, *p* = 0.016). In contrast, the fraction of time spent in State 2 was negatively correlated with epilepsy duration (Figure 7B, *r* = −0.51, *p* = 0.016). Furthermore, the MDT in State 1 was positively correlated with epilepsy duration (Figure 7C, *r* = 0.67, *p* = 0.001). Additionally, we found negative (but not significant) associations between the MDT in State 2 and epilepsy duration (Figure 7D, *r* = −0.38, *p* = 0.08).

## 4. Discussion

Recently, dFC has increasingly been applied in the study of epilepsy to capture time-varying properties. In this study, we employed resting-state fMRI with ICA, sFC, and dFC analysis to investigate alterations in whole-brain networks in children with GTCS. Our analysis revealed the following: (1) Both the sFC and dFC of the GTCS children’s whole-brain network were changed significantly. (2) We compared the sFC and dFC profiles and found that there was a shared decrease in connectivity across both methods and some specific connections by each method. Interestingly, children with GTCS showed a significant increase in their dFC between the subnetworks in State 2 only. (3) Regarding the dynamic properties of the functional brain network, children with GTCS had fewer occurrences and a shorter MDT in State 1 and more occurrences and a longer MDT in State 2. These changes in dFC properties were linked to epilepsy duration. In summary, these results may support the hypothesis that children with GTCS exhibit temporal variability in whole-brain network connectivity. Combining the sFC and dFC methods might provide more valuable information regarding the neural mechanism and possible neuroimaging biomarkers of clinical diagnoses in children with GTCS epilepsy.

### 4.1. Group differences in sFC analysis between the whole-brain subnetworks

Epilepsy is considered to disrupt neural networks (Royer et al., 2022). Compared to HCs, children with GTCS exhibited significant abnormal sFC among resting-state subnetworks. Our results showed that the decreased sFC of GTCS children mainly affected internetwork connectivity among the VIS, SMN, DMN, FPN, Lim, and VAN. The SMN is a motor network that is also integrated into a multimodal network associated with motor systems and cognitive hub regions (Sepulcre et al., 2012). The FPN is highly integrated with other brain networks associated with cognitive control, providing a functional backbone for rapid and flexible modulation of other brain networks (Marek and Dosenbach, 2018). The DMN is the most important brain network for the modulation of internal mentation and external cognitive processes (Raichle et al., 2001). It has been reported that temporal lobe epilepsy patients showed slower spreading time in FPN, Lim, DMN, and subcortical networks with impairment in sensorimotor, executive, and memory functions (Girardi-Schappo et al., 2021). Whole-brain network dysfunction may contribute to cognitive impairments in epilepsy patients. In patients with idiopathic generalized epilepsy, significant decreases in sFC of the bilateral medial prefrontal cortex and precuneus/posterior cingulate cortex were observed (Kim et al., 2014). Altered thalamocortical functional connectivity may be the long-term consequence of epilepsy (Wang et al., 2012). All these previous studies showed that inter-network connectivity patterns may be important in maintaining normal cognitive functions. A possible explanation of cognitive impairment in patients with epilepsy may be related to the impaired functional integrations between these subnetworks. In the present study, the sFC among the FPN, SMN, DMN, and VIS showed a significant decrease. These results are consistent with the main results of previous studies on epilepsy patients and indicate that the pathway of visual information projections from the visual cortex to the FPN and SMN may be inhibited in epilepsy patients. We also observed a significant decrease in the sFC between the DMN and other subnetworks (the SMN, Lim, and VIS). Considering the important role of the DMN in the brain, a reduction in connectivity strength between the DMN and other subnetworks may imply that information transmission and efficiency are suppressed in children with GTCS. Based on graph theory analysis, previous studies have reported that patients with GTCS exhibit significant reductions in rich-club connectivity among central hubs (Li et al., 2016). Long-term damage due to epilepsy may disrupt rich-club organization. As a result, GTCS patients showed reduced brain integration among different functional domains. Another perspective that main explain the significant decrease in sFC in children with GTCS may be altered functional-structural coupling of large-scale brain networks in GTCS patients (Zhang et al., 2011; Liao et al., 2013; Liao et al., 2016). Previous neuroimaging studies have reported widespread and subtle changes in gray matter volume (Ciumas and Savic, 2006; Allen et al., 2020). Long-term and progressive remodeling of the brain structure in patients with GTCS may induce changes in the brain’s functional architecture given the relationship between function and structure. Based on the above discussion, the observations in the present study may indicate abnormal functional relationship among these subnetworks in child patients. GTCS could mainly impact internetwork communications in children.

Regarding the sFC results, we should note that no significant increases in sFC were detected in children with GTCS. This result is not consistent with that of previous studies on adult patients with GTCS. A previous study using a similar method found that adults with GTCS demonstrated a significant increase in the sFC among the DMN, FPN, and attention network (Wei et al., 2015). A recent study in patients with generalized epilepsy also showed both an increase and decrease in activation patterns among the DMN, motor cortex, and attention network (Klamer et al., 2018). The possible reason for the above inconsistency may be the difference in the age of the research subjects. These previous studies focused on adults with GTCS. In the present study, we focused on the changes in FC patterns in children with GTCS. Chronic epilepsy may disrupt brain organization and normal brain development in children with GTCS. As a result, abnormal functional connectivity among whole-brain subnetworks may stem from the combined effects of epileptic seizures and brain development. Children with GTCS exhibited a specific difference in connectivity patterns compared with those of adult patients. Our previous research on children with GTCS also showed a significant decrease in connectivity of the thalamocortical network (Li et al., 2022c) and a significant decrease in spontaneous brain activity in the bilateral angular gyrus, left inferior, and middle temporal gyrus (Wang et al., 2018). Thus, the sFC among the whole-brain subnetworks in the present study further confirmed the view that children with GTCS show a specific connectivity pattern.

### 4.2. Group differences in dFC analysis among the whole-brain subnetworks

Although we did not detect a significant increase in brain connectivity with the sFC method, investigating the functional interactions between the resting-state networks in terms of dFC provided additional information regarding the neural mechanism of GTCS in the present study (specifically, we observed both increases and decreases in connectivity). Recently, the dynamic functional brain connectome has been recognized as a novel approach to tracking the dynamics of the mental state (Chang and Glover, 2010; Allen et al., 2014). In addition to sFC, we investigated brain abnormalities in functional network by the dFC method. Two recurring states were identified in the functional MRI scan. State 1 was characterized by hyperconnectivity across the whole brain. State 2 was characterized by weak connections across most brain networks. In each state, children with GTCS showed significant differences in dFC strength compared with HCs. In State 1, children with GTCS showed a significant decrease in the dFC strength between the VIS and other cognitive networks (the FPN and SMN). The change direction of dFC and the main networks with altered dFC in State 1 were consistent with the sFC results. Both sFC and dFC decreases in inter-network connectivity may reflect the chronic effect of epilepsy on brain functional organization in children with GTCS.

Another main finding regarding altered connectivity in the present study was that inter-network connections between most subnetworks showed a significant increase in the dFC in State 2. As shown in Figure 5B, significant between-group dFC differences in State 2 were observed among several subnetworks including the DMN, DAN, VAN, Lim, FPN, and VIS. Attention is the basis for higher cognition. Both the DAN and VAN are part of the attention system. The DAN mediates top-down attention, and the VAN mediates bottom-up attention. Our results showed that the dFC strengths between attention networks and cognitive networks (the FPN and VIS) were significantly enhanced in children with GTCS in State 2. Regarding attention networks, previous studies have shown that seizure activity in epilepsy may disturb brain networks and damage brain areas associated with attention (Jiang et al., 2018; Wu et al., 2021). Damage to the attention network may suppress the ability to disengage from the current target of attention. As a result, attention is impaired in children with epilepsy, and the connectivity of attention-related networks is disrupted. To achieve the same cognitive performance as HCs, epilepsy patients need to devote more resources (or pay more attention) to information transmission in attention networks regarding the external environment. Thus, inter-network connectivity (between the attention networks and FPN/VIS) showed a significant increase in children with GTCS.

It is worth noting that the increased inter-network connectivity in State 2 was mainly related to the DMN (DMN vs. VAN, DMN vs. DAN, DMN vs. VIS). The DMN has been confirmed to integrate information from primary sensory areas and cognitive networks (Buckner and DiNicola, 2019). Previous studies in patients with GTCS have frequently reported a decrease in the connective patterns within the DMN and an increase in the connective patterns between the DMN and the other cognitive networks (Li et al., 2020a,2022a; Parsons et al., 2020). In addition, a previous study found a significant increase in the dFC strengths between the DMN and VIS (Liu et al., 2017). Recently, increased FC variabilities were also detected between the DMN and cognition-related networks (the VAN, DAN, and FPN) in GTCS (Jia et al., 2020). These inter-networks results might lead to functional confusion of the task-negative (DMN) and task-positive networks (the VAN, DAN, FPN). Our dFC results in State 2 are consistent with these previous reports and indicated that disruption of whole-brain information transmission is associated with cognitive impairments in patients with GTCS.

Comparison of the sFC and dFC results revealed that the decreased inter-network connections were shared by both methods, but the increased inter-network connections were only detected by the dFC method. The reason for this may be that sFC represents of the average connectivity across different dynamic states throughout the whole scanning period but does not reflect the time-dependent and dynamic nature of brain activity. The dynamic FC method may be more sensitive than the sFC method to capture the between-group alteration (Rolls et al., 2021). The findings of the present study further confirm that the dFC approach can provide additional information concerning the brain’s functional organization in epilepsy patients. The present results indicate that combining the sFC and dFC can provide complementary information regarding brain organization in children with GTCS.

The present findings demonstrated that inter-network dFC between subnetworks in children with GTCS showed a significant increase in State 2 and a decrease in State 1 as compared to the control group. Such direction of change are consistent with previous findings that children with GTCS showed both an increase and decrease in dFC strength of some specific networks (Li et al., 2022a,c). However, previous studies on adults with GTCS only detected increased FC variabilities between the DMN and cognition-related networks (Jia et al., 2020; Yang et al., 2021). A possible explanation for the inconsistency in results between the present study and previous studies in adults with GTCS may be the differences in the research subjects. Previous studies have demonstrated that children with GTCS showed a different brain organization than adults with GTCS (Li et al., 2016, 2017, 2020a; Liu et al., 2017). In addition to epilepsy seizures, brain development may play a greater role in children with epilepsy than in adults with epilepsy. The above explanations should be examined in future studies with improved designs, including both adults and children with GTCS.

### 4.3. Significant correlations between dFC properties and epilepsy duration in children with GTCS

Regarding the temporal properties, (dwell time and fractional windows), both decreased significantly in State 1 and increased significantly in State 2 in children with GTCS. High connectivity and short mean dwell time were detected in State 1 and low connectivity and long mean dwell time were detected in State 2. The changes in the directions of these dFC properties are consistent with previous dingings in patients with GTCS (Liu et al., 2017; Li et al., 2022a). We speculated that the opposite changes observed in the temporal properties of the two states might lead to disrupted communication among resting-state networks. And also, there were significant correlations between these two properties and epilepsy duration in both states. Previous studies on epilepsy patients have found that brain functional organization, such as dFC variability and dFC temporal properties, can be affected by epilepsy duration (Jia et al., 2020; Li et al., 2022a). These present correlation findings emphasized the importance of epilepsy duration for the brain’s functional architecture. Dynamic FC analyses could yield additional information regarding GTCS in children.

### 4.4. Limitations

Our study also has several limitations. First, the sample size was relatively small, with just 55 participants (23 patients and 32 HCs), although this size was sufficient for the statistical analyses. A larger sample size, especially more patients, should be used in future studies to investigate the replicability of the present findings. Second, this study was a cross-sectional study, which prevents determination of causality in the relationship between FC alterations and disease progression. Future studies should used a longitudinal design to examine this relationship. Third, the present study focused on gray matter signals. The signals within the white matter were excluded. A recent study found that the resting-state fMRI signals in white matter may also vary with physiological states (Basile et al., 2022). Future studies should take white matter signals into account in epilepsy patients. Fourth, all children with GTCS were medicated in the present study. Using antiepileptic drugs can affect normal neuronal function and induce cognitive impairment in children (Ijff and Aldenkamp, 2013). Future studies should consider the effect of antiepileptic drugs on brain connectivity in children with GTCS. Finally, some children under the age of 4 years were sedated during image collection, and sedative use may also affect brain connectivity. Future studies are needed to clarify the effect of sedative drugs on brain connectivity.

## 5. Conclusion

In the present study, we employed ICA, sFC, and dFC analyses of resting-state fMRI data to investigate the alterations in whole-brain networks in children with GTCS. Both the sFC and dFC of the whole-brain networks in children with GTCS showed significant changes. In particular, both increase and decrease in dFC were detected among whole-brain resting-state networks, while decreased sFC was observed. There was temporal variability in brain network connectivity in children with GTCS. We also found significant correlations between the temporal properties of dFC and epilepsy durations. These results demonstrated significant alterations in connectivity strength and temporal properties of dFC in the whole-brain network in children with GTCS. These findings can enhance understanding of the neural mechanisms underlying GTCS in children. This study further confirms that investigating brain functional architectures from static and dynamic perspectives can provide more comprehensive insight into the abnormal changes in brain network connectivity in children with epilepsy.

## Data availability statement

The raw data supporting the conclusions of this article will be made available by the authors, without undue reservation.

## Ethics statement

The studies involving humans were approved by the Ethical Committee of Shenzhen Children’s Hospital. The studies were conducted in accordance with the local legislation and institutional requirements. Written informed consent for participation in this study was provided by the participants’ legal guardians/next of kin. Written informed consent was obtained from the individual(s), and minor(s)’ legal guardian/next of kin, for the publication of any potentially identifiable images or data included in this article.

## Author contributions

YL and QC conceived and designed the experiments. QC performed the experiments. YL analyzed image data, sorted the results, and wrote and reviewed the manuscript. YR and YL were responsible for patient management and conceptualized the study. All authors contributed to the article and approved the submitted version.

## Footnotes

1
http://mialab.mrn.org/software/gift

2
https://surfer.nmr.mgh.harvard.edu/fswiki/CorticalParcellation_Yeo2011

3
www.restfmri.net/forum/DynamicBC
