# Supplementary material for: Abnormal static and dynamic functional network connectivity of the whole-brain in children with generalized tonic-clonic seizures
Source: Front Neurosci. 2023 Aug 21;17:1236696. doi: 10.3389/fnins.2023.1236696 (PMC10475552; doi:10.3389/fnins.2023.1236696)
Supplement: Supplementary file 1 [file Data_Sheet_1.docx]

***Supplementary Material***

**Supplementary Figures**

To verify the stability of dFC results, we also selected a sliding window with a size of 40 TRs and repeated the above analysis. Similar results were obtained by using different window lengths. Supplementary Figure 1-3 showed the dFC results of the window length of 40 TRs.

**
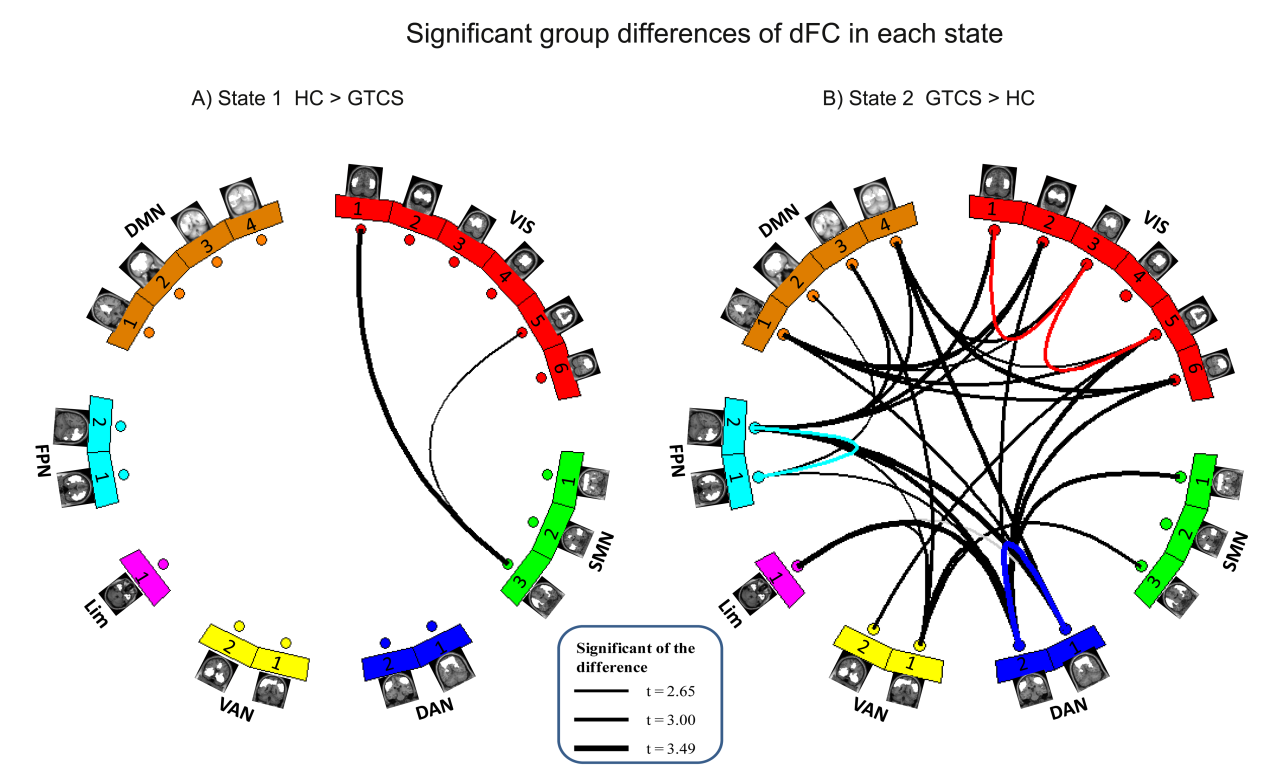
**

**Supplementary Figure 1.** Group differences of dynamic functional network connectivity between two groups in seven functional networks. Children with GTCS had weaker dFC pathways in State 1 (A) and stronger dFC pathways in State 2 (B) as compared with the HC groups.

DAN = dorsal attention network, VAN = ventral attention network, Lim = limbic network, FPN = frontoparietal network, DMN = default mode network.

**
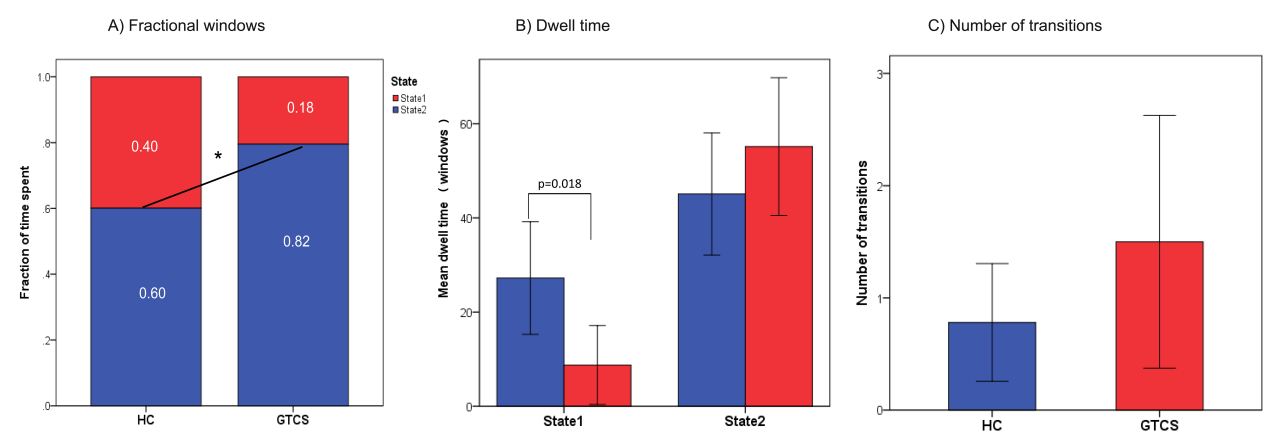
**

**Supplementary Figure 2.** Temporal properties of the dynamic states.

(A) The mean fractional properties spent in each state are different between groups. (B) The mean dwelling time in State 1 is shorter in GTCS children than in controls. (C) The number of transitions between the two groups.

* Indicates a significant difference between the two groups.

GTCS = generalized tonic-clonic seizures children, HC = healthy control.

**
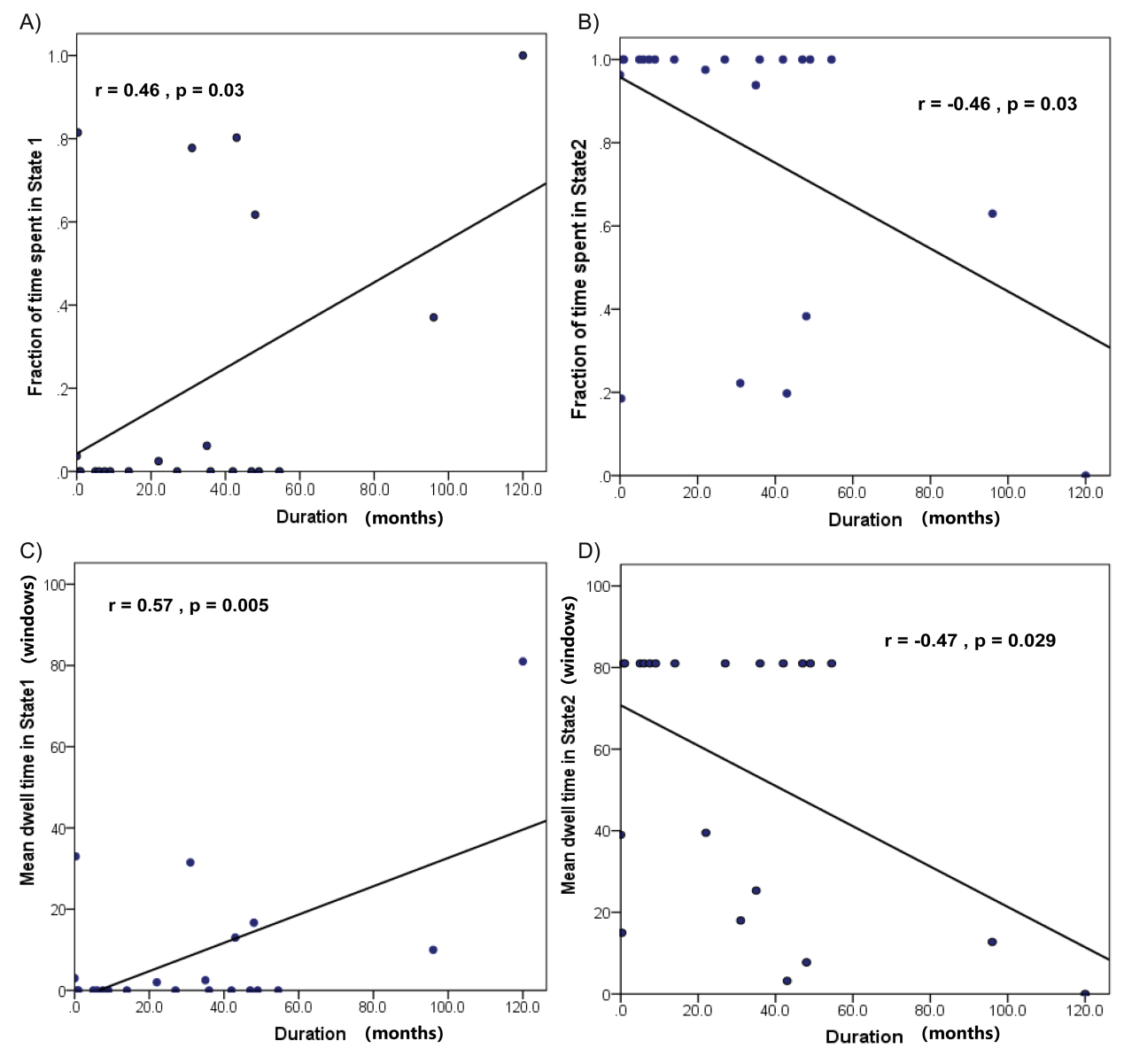
**

**Supplementary Figure 3.** Correlation between the temporal properties of dFC and the epilepsy durations in GTCS children.

(A) The fraction of time spent in State 1 is positively correlated with the epilepsy durations. (B) The fraction of time spent in State 2 is negatively correlated with epilepsy durations. (C) The mean dwell time in State 1 is positively correlated with the epilepsy durations. (D) The mean dwell time in State 2 is not significantly correlated with the epilepsy durations.
